# Supplementary material for: Multi‐dataset identification of innovative feature genes and molecular mechanisms in keratoconus
Source: J Cell Mol Med. 2024 Sep 19;28(18):e70079. doi: 10.1111/jcmm.70079 (PMC11412914; doi:10.1111/jcmm.70079)
Supplement: Supplementary file 2 — Material S2. [file JCMM-28-e70079-s002.zip › 7.GSEA analysis/3.DBNDD1/KEGG_CYTOSOLIC_DNA_SENSING_PATHWAY.html]

Details for gene set KEGG\_CYTOSOLIC\_DNA\_SENSING\_PATHWAY[GSEA]

|  || Dataset | DBNDD1.group.cls#h\_versus\_l |
| Phenotype | group.cls#h\_versus\_l |
| Upregulated in class | l |
| GeneSet | KEGG\_CYTOSOLIC\_DNA\_SENSING\_PATHWAY |
| Enrichment Score (ES) | -0.35125273 |
| Normalized Enrichment Score (NES) | -1.1856637 |
| Nominal p-value | 0.24493927 |
| FDR q-value | 0.7253733 |
| FWER p-Value | 0.99 |
Table: GSEA Results Summary

  

Fig 1: Enrichment plot: KEGG\_CYTOSOLIC\_DNA\_SENSING\_PATHWAY      
 Profile of the Running ES Score & Positions of GeneSet Members on the Rank Ordered List

  

| SYMBOL | TITLE | RANK IN GENE LIST | RANK METRIC SCORE | RUNNING ES | CORE ENRICHMENT || 1 | IFNA14 | na | 550 | 0.438 | 0.0735 | No |
| 2 | IFNA6 | na | 1774 | 0.315 | 0.1025 | No |
| 3 | IFNA16 | na | 4203 | 0.207 | 0.0746 | No |
| 4 | PYCARD | na | 8952 | 0.086 | -0.0446 | No |
| 5 | RIPK1 | na | 8963 | 0.086 | -0.0273 | No |
| 6 | CASP1 | na | 9653 | 0.076 | -0.0317 | No |
| 7 | POLR3H | na | 9839 | 0.073 | -0.0220 | No |
| 8 | POLR3B | na | 10585 | 0.063 | -0.0306 | No |
| 9 | MAVS | na | 12871 | 0.039 | -0.0884 | No |
| 10 | IFNA2 | na | 13108 | 0.037 | -0.0876 | No |
| 11 | ZBP1 | na | 14600 | 0.026 | -0.1253 | No |
| 12 | POLR1C | na | 14659 | 0.025 | -0.1219 | No |
| 13 | POLR3K | na | 15696 | 0.018 | -0.1481 | No |
| 14 | IFNB1 | na | 15923 | 0.016 | -0.1513 | No |
| 15 | POLR3A | na | 16649 | 0.012 | -0.1698 | No |
| 16 | CCL4L2 | na | 16968 | 0.010 | -0.1770 | No |
| 17 | TBK1 | na | 16997 | 0.009 | -0.1759 | No |
| 18 | TREX1 | na | 17916 | 0.003 | -0.2017 | No |
| 19 | POLR3GL | na | 17941 | 0.003 | -0.2018 | No |
| 20 | IL18 | na | 18323 | 0.001 | -0.2127 | No |
| 21 | ADAR | na | 18359 | 0.000 | -0.2136 | No |
| 22 | CHUK | na | 18953 | -0.004 | -0.2299 | No |
| 23 | IRF7 | na | 19009 | -0.005 | -0.2305 | No |
| 24 | POLR1D | na | 20038 | -0.013 | -0.2575 | No |
| 25 | AIM2 | na | 20470 | -0.017 | -0.2664 | No |
| 26 | POLR3F | na | 20850 | -0.021 | -0.2731 | No |
| 27 | NFKBIB | na | 21812 | -0.030 | -0.2947 | No |
| 28 | IFNA4 | na | 22069 | -0.033 | -0.2954 | No |
| 29 | IKBKB | na | 22300 | -0.035 | -0.2948 | No |
| 30 | POLR3D | na | 22729 | -0.040 | -0.2990 | No |
| 31 | IKBKG | na | 23063 | -0.044 | -0.2997 | No |
| 32 | IL6 | na | 24766 | -0.067 | -0.3350 | Yes |
| 33 | IKBKE | na | 25105 | -0.072 | -0.3300 | Yes |
| 34 | NFKB1 | na | 25842 | -0.084 | -0.3340 | Yes |
| 35 | RIPK3 | na | 26019 | -0.088 | -0.3212 | Yes |
| 36 | IRF3 | na | 26100 | -0.089 | -0.3053 | Yes |
| 37 | POLR3G | na | 26624 | -0.100 | -0.2999 | Yes |
| 38 | NFKBIA | na | 27480 | -0.118 | -0.3005 | Yes |
| 39 | IL33 | na | 28430 | -0.142 | -0.2989 | Yes |
| 40 | RELA | na | 28616 | -0.147 | -0.2744 | Yes |
| 41 | IL1B | na | 28762 | -0.150 | -0.2479 | Yes |
| 42 | IFNA8 | na | 30493 | -0.203 | -0.2563 | Yes |
| 43 | IFNA7 | na | 30811 | -0.214 | -0.2217 | Yes |
| 44 | CCL5 | na | 32225 | -0.277 | -0.2059 | Yes |
| 45 | IFNA10 | na | 32985 | -0.328 | -0.1609 | Yes |
| 46 | IFNA21 | na | 34135 | -0.449 | -0.1025 | Yes |
| 47 | CXCL10 | na | 34557 | -0.586 | 0.0049 | Yes |
Table: GSEA details [plain text format]

  

Fig 2: KEGG\_CYTOSOLIC\_DNA\_SENSING\_PATHWAY      
 Blue-Pink O' Gram in the Space of the Analyzed GeneSet

  

Fig 3: KEGG\_CYTOSOLIC\_DNA\_SENSING\_PATHWAY: Random ES distribution      
 Gene set null distribution of ES for **KEGG\_CYTOSOLIC\_DNA\_SENSING\_PATHWAY**
